# Multi-Tracer PET-Guided Therapy Selection and Theranostics in Radioiodine-Refractory Thyroid Cancer Patient Unsuitable for Tyrosine Kinase Inhibitor Therapy

**DOI:** 10.1055/s-0045-1812056

**Published:** 2025-10-04

**Authors:** Austin Saju, Sunita Sonavane, Sandip Basu

**Affiliations:** 1 Radiation Medicine Centre, Bhabha Atomic Research Centre, Tata Memorial Hospital Annexe, Jerbai Wadia Road, Parel, Mumbai, Maharashtra, India; 2 Homi Bhabha National Institute, Mumbai, Maharashtra, India

**Keywords:** radioiodine-refractory thyroid cancer, multi-tracer PET/CT, theranostics, targeted radionuclide therapy, molecular imaging, PET-guided treatment planning

## Abstract

Radioactive iodine refractory (RAIR) thyroid cancer and thyroglobulin elevated negative iodine scintigraphy constitute a fraction of thyroid cancer patients with poorer prognosis as opposed to radioiodine concentrating disease. Although not a rare condition and with several treatment options already explored, the selection of an optimal therapy that balances effective disease stabilization, symptom palliation, life prolongation, and minimal side effects remains essential. The use of multi-tracer positron emission tomography (PET) shows potential for radionuclide therapy selection in RAIR thyroid cancer. In this case report, multi-tracer PET imaging was employed to assess the feasibility of available theranostic options. While [ ^68^ Ga]Ga-DOTATATE PET/computed tomography (CT) showed low-level somatostatin receptor expression, [ ^68^ Ga]Ga-prostate specific membrane antigen (PSMA)-11 PET/CT revealed moderate and concordant PSMA expression across all lesions observed on [ ^18^ F]F-FDG PET/CT, justifying [ ^177^ Lu]Lu-PSMA-617 therapy in this patient.

## Introduction

Recent data ranks thyroid carcinoma as the 16th most common cancer among males and fifth among females, with differentiated thyroid carcinoma (DTC) accounting for 90% of cases. 1 DTC has a favorable 10-year survival of 90%. Standard treatment includes surgery, thyroid-stimulating hormone (TSH) suppression, and radioactive iodine (RAI) therapy. 1 2 Approximately 10 to 15% of cases develop distant metastasis necessitating directed or other systemic therapy. 3 Long-term outcomes depend on treatment success, including surgical completeness, RAI therapy efficacy, and additional therapies like external beam radiotherapy (EBRT) and/or systemic therapy. 1 However, around 5 to 15% of patients demonstrate radioactive iodine refractory (RAIR) disease at initial therapeutic radioiodine scintigraphy or after adjuvant therapy in advanced cases, which confers a poor prognosis. 1 2 The inability to treat further with RAI prompts a different approach to therapy—mostly for pain palliation and improving quality of life. Limited treatment response and/or significant debilitating side effects of alternative therapeutic options necessitate careful therapy selection and sequencing, with metabolic and receptor-based positron emission tomography (PET) imaging playing a key role, as illustrated in the following case.

## Case Report

**Fig. 1:**
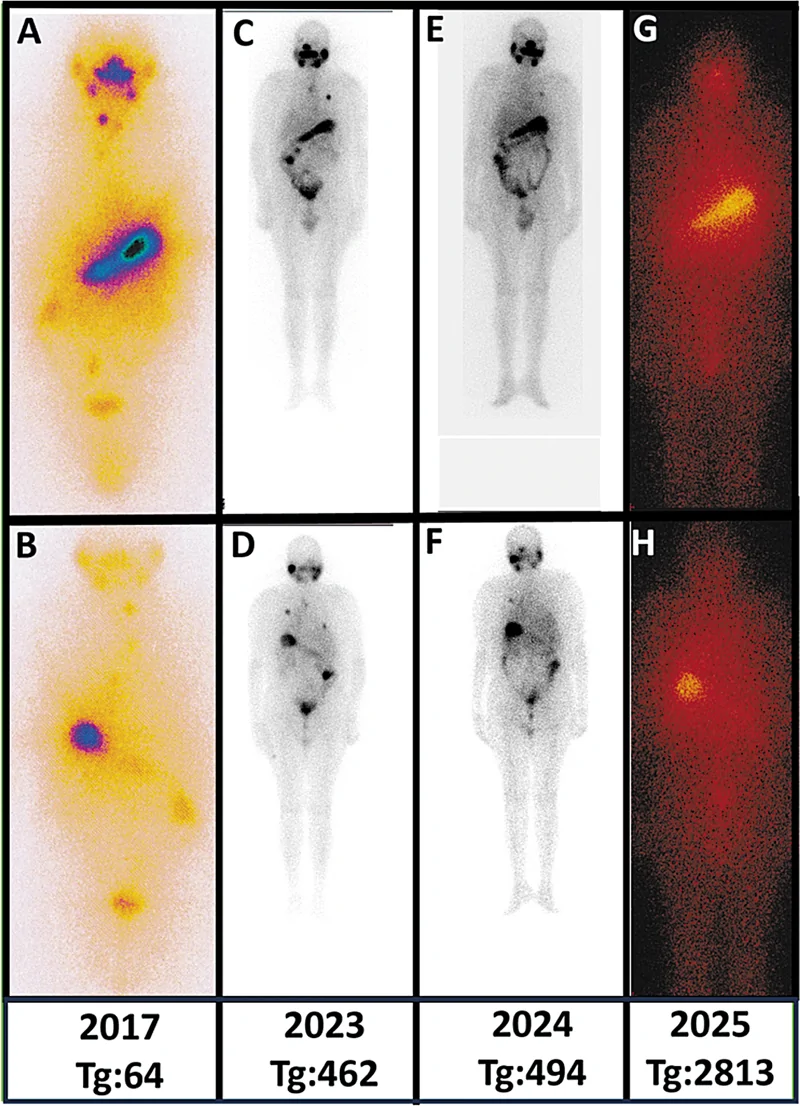
Whole-body RAI planar scans in anterior (upper panel) and posterior (lower panel) views at various time points, along with corresponding serum thyroglobulin (Sr Tg) values (ng/dL). ( **A, B** ) First posttherapy scan (2017) showing remnant neck uptake. ( **C–F** ) Subsequent post-RAI therapy scans in 2023 and 2024 demonstrate RAI-avid C2 vertebral lesion and lung nodules, with elevated Sr Tg levels. ( **G, H** ) Whole-body diagnostic scan in 2025 showing no RAI-avid foci and a significant increase in Sr Tg.

**Fig. 2:**
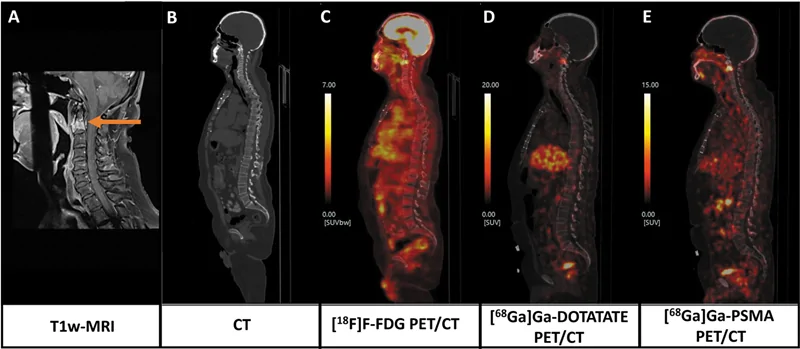
Sagittal views demonstrating a metastatic lytic lesion in the C2 vertebra. ( **A** ) Sagittal T1-weighted MRI shows a hyperintense lytic lesion in the C2 vertebral body (orange arrow). ( **B** ) CT image reveals osteolytic destruction of the C2 vertebra. ( **C** ) Multitracer PET scan shows moderate [ ^18^ F]FDG uptake in the lesion (SUV _max_ : 8.5). ( **D** ) Mild somatostatin receptor (SSTR) expression is observed (Krenning score 2; SUV _max_ : 9.8). ( **E** ) Moderate PSMA expression is noted (miPSMA score 2; SUV _max_ : 19.2).

**Fig. 3:**
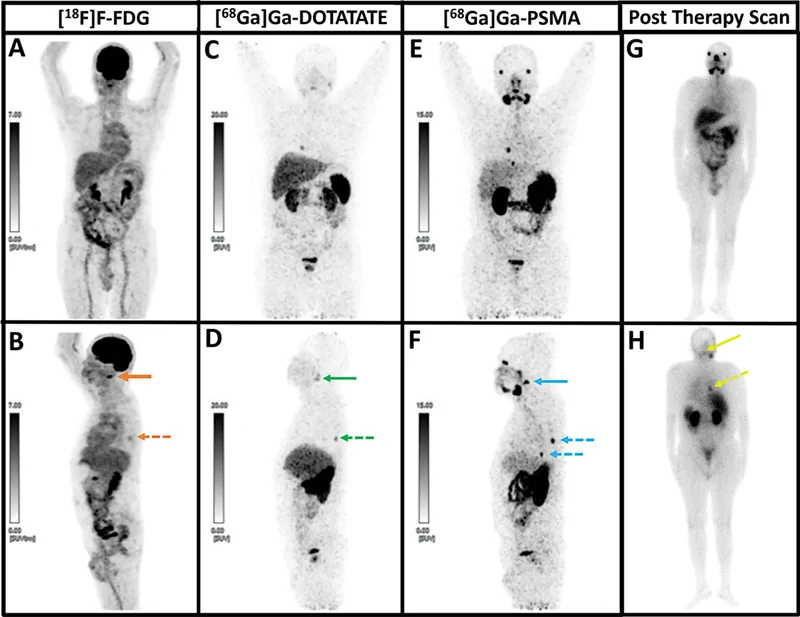
Maximum intensity projection (MIP) images from three radiotracers. ( **A, B** ) [ ^18^ F]FDG PET showing moderate uptake in the C2 vertebra (solid orange arrow) and low-grade uptake in a solitary lung nodule (broken orange arrow). ( **C, D** ) [ ^68^ Ga]DOTATATE PET showing mild somatostatin receptor expression (Krenning score 2) in both the C2 vertebral lesion (solid green arrow) and the lung nodule (broken green arrow). ( **E, F** ) [ ^68^ Ga]PSMA-11 PET demonstrating moderate PSMA expression (miPSMA score 2) in the C2 vertebral lesion (solid blue arrow) and two lung nodules (broken blue arrows).

A 72-year-old hypertensive male with low-risk follicular thyroid carcinoma underwent total thyroidectomy and 30 mCi RAI ablation in 2017 ( Fig. 1A, B ). Four years later, he presented with cervical neck pain and elevated serum thyroglobulin (Sr Tg) of 264.1 ng/mL. A whole-body diagnostic RAI scintigraphy showed RAI uptake in a lytic C2 vertebral lesion and sub-centimetric lung nodules, with moderate and low-grade [ ^18^ F]F-FDG uptake on whole body [ ^18^ F]F-FDG PET/computed tomography (CT). He received EBRT to the C2 vertebra and two cycles of RAI with 100 mCi (2023) and 150 mCi (2024; Fig. 1C–F ). One year posttreatment, he had mild persisting neck pain and an abrupt rise in Sr Tg 2568 ng/mL with anti-thyroglobulin antibody (ATA) of 23.9 IU/mL (normal < 4.1 IU/mL), and negative RAI scintigraphy ( Fig. 1G, H ) despite adequate iodine restriction, qualifying as a case of thyroglobulin elevated negative iodine scintigraphy syndrome. Restaging [ ^18^ F]F-FDG-PET/CT showed moderate uptake in the C2 lesion (SUV _max_ : 8.5; Fig. 2C ) and low-grade uptake in two lung nodules (SUV _max_ : 3.1–4.3; Fig. 3A, B ). Given his age, limited disease, expected morbidity from a hypermetabolic lytic C2 lesion, and low metabolism in the lung nodules, the therapeutic options considered were surgical debridement with fixation of the C2 lesion, repeat EBRT, and addition of tyrosine kinase inhibitor (TKI) with continued TSH suppression. However, the patient denied surgery. Empirical RAI was ruled out, as structural disease with potential morbidity was evident. Although TKIs are well-established in such cases, considering his current well-being, cardiac comorbidities, limited disease, potential side effects of TKI, and only a single C2 lesion out of three metastatic sites showing moderate ^18^ F-FDG uptake, other possible targeted radionuclide therapies (TRT) were explored. Staging PET/CT with [ ^68^ Ga]Ga-DOTATATE showed low somatostatin receptor (SSTR) expression (Krenning score 2) in two out of three lesions ( Fig. 3C, D ). In contrast, [ ^68^ Ga]Ga-prostate specific membrane antigen (PSMA)-11 PET/CT revealed moderate PSMA expression in all lesions (miPSMA score 2; Fig. 3E, F ) as highlighted in Table 1 , supporting the use of [ ^177^ Lu]Lu-PSMA-617 TRT. He was treated with one cycle of 6.6GBq [ ^177^ Lu]Lu-PSMA-617. On 6 weekly follow-ups, he reported a significant reduction of neck pain, no clinical signs of radiculopathy, and serial reduction of Sr. Tg (249 ng/mL) and ATA (9.52 IU/mL, normal < 4.11 IU/mL).

**Table 1:** Maximum standard uptake value (SUV _max_ ) of lesions in multi-tracer PET

Lesion	[18F]F-FDG	[68Ga]Ga-DOTATATE	[68Ga]Ga-PSMA
C2 vertebral lesion	8.60	9.83 [Krennings 2]	19.20 [miPSMA 2]
Right lung lower lobe medial basal	3.89	8.80 [Krennings 2]	22.90 [miPSMA 2]
Right lung lower lobe posterior basal	2.50	8.42 [Krennings 2]	20.30 [miPSMA 2]

## Discussion

Approximately 5 to 15% of thyroid carcinoma patients show RAIR disease at initial posttherapeutic radioiodine scintigraphy or after adjuvant therapy in advanced cases. 1 2 RAIR entails a poor prognosis, with some patients dying within 3 to 5 years, while the remaining have progressive, slow-growing disease. 1 Therapeutic decision making can be challenging, requiring consideration of the age of the patient, life expectancy, comorbidities, symptoms, disease burden by imaging (CT, magnetic resonance imaging [MRI], or [ ^18^ F]F-FDG PET/CT), and the rate of progression of disease either radiologically or biochemically. Watchful observation with continued TSH suppression for stable or slow-growing low-volume disease can be considered due to the lack of effective, tolerable therapies with minimal side effects. However, active treatment must be sought for progressive disease, pain palliation, or prevention of morbidity. 1 Therapeutic options include directed treatments like surgery, EBRT, and thermal ablation, or systemic approaches such as empiric RAI, TKI (e.g., lenvatinib and sorafenib), TRT (e.g., [ ^177^ Lu]Lu-DOTATATE, [ ^177^ Lu]Lu-PSMA, and [ ^177^ Lu]Lu-FAPI), though the latter are viable in limited cases. Empirical RAI is generally recommended for biochemically suspected but structurally occult disease, aiming to localize treatable lesions or manage unresectable ones. While 50% show a biochemical Tg decline, it offers no survival benefit. The greatest anatomical response is seen in pulmonary metastases. 1 The landmark SELECT and DECISION trials showed improved progression-free survival with lenvatinib (18.3 months) and sorafenib (10.8 months) in progressive RAIR thyroid cancer. 4 5 However, side effects led to treatment discontinuation in approximately 20% of patients. 1

An alternate therapy is TRT with β-particle-emitting [ ^177^ Lu]Lutetium-labeled peptides. Approved TRTs are [ ^177^ Lu]Lu-DOTATATE for SSTR-expressing gastroenteropancreatic neuroendocrine tumors and [ ^177^ Lu]Lu-PSMA-617 for PSMA-expressing metastatic castration-resistant prostate carcinoma (mCRPC) due to demonstrated efficacy, tolerability, and low side-effect profile in multicentric trials. 6 7 8 However, PSMA is not entirely tumor-specific. While it is highly overexpressed on tumor cells in mCRPC, PSMA expression can also be observed in the neovasculature of various other malignancies, including metastasis from thyroid carcinoma. 9 Though multiple studies evaluating the role of radio-labeled PSMA PET imaging in RAIR thyroid carcinoma have been conducted, scarce literature is available for PSMA radioligand therapy in a similar setting. Our literature review yielded only two such related articles. Vreis et al published a case series of five patients with RAIR thyroid carcinoma treated with [ ^177^ Lu]Lu-PSMA therapy and reported modest or equivocal responses, with transient Sr Tg decline followed by disease progression after a few months. 2 Assadi and Ahmadzadehfar reported a RAIR thyroid carcinoma patient treated sequentially with [ ^177^ Lu]Lu-DOTATATE and [ ^177^ Lu]Lu-PSMA, who died from cardiac arrest 2 weeks post-[ ^177^ Lu]Lu-PSMA, precluding any further clinical follow-up. 10

A notable consideration in this case was the discordant biological behavior between the lytic C2 vertebral lesion and the lung nodules, as reflected by varying [ ^18^ F]F-FDG uptake. The aggressive, symptomatic C2 lesion could have been surgically managed, while the indolent pulmonary nodules, exhibiting low metabolic activity, were potential candidates for monitoring under TSH suppression. However, the patient's refusal of surgery and continued disease progression despite EBRT necessitated exploration of further treatment options. Although TKI therapy is the conventional next step for progressive RAIR disease, in this case, several factors weighed against its immediate initiation: the patient's relatively preserved performance status, cardiac comorbidities, limited disease burden, and anticipated toxicity associated with TKI use. Consequently, the clinical team considered TRT as an interim approach, aiming to delay the introduction of TKI and reduce treatment-related morbidity. Multi-tracer PET imaging was employed to assess suitability for theranostic approaches. While [ ^68^ Ga]Ga-DOTATATE PET/CT showed low-level SSTR expression, [ ^68^ Ga]Ga-PSMA-11 PET/CT revealed moderate and concordant PSMA uptake across all lesions, supporting the use of [ ^177^ Lu]Lu-PSMA-617 therapy in this patient. Although no standardized guidelines currently exist for the implementation of TRT in RAIR disease, multi-tracer PET-guidance for TRT is being considered at an institutional level for patients with progressive RAIR disease who have no other definitive therapeutic options. 11 Such patients are enrolled in a research study to evaluate the potential for TRT, or, in very advanced cases, considered on compassionate grounds.

## Conclusion

TRT represents a well-tolerated, viable treatment option in selected RAIR thyroid carcinoma patients, particularly in those with clinically stable, low-burden disease with favorable uptake on diagnostic PET imaging. Therapy sequencing based on multi-tracer PET/CT provides valuable insight, potentially allowing deferral of TKI therapy and its associated toxicity. While evidence supporting [ ^177^ Lu]Lu-PSMA-617 in RAIR thyroid cancer remains preliminary, its utility in carefully selected cases, like the one presented, merits further clinical investigation.
